# Is There Any Role for D3 Lymphadenectomy in Gastric Cancer?

**DOI:** 10.3389/fsurg.2018.00027

**Published:** 2018-03-22

**Authors:** Gerassimos N. Douridas, Stefanos K. Pierrakakis

**Affiliations:** ^1^Department of General Surgery, Thriasio General Hospital, Athens, Greece

**Keywords:** D3, lymphadenectomy, gastric, cancer, surgery, extend, lymph nodes, gastrectomy

## Abstract

Although D2 constitutes the level of lymph node dissection which most surgical associations endorse in their treatment guidelines for gastric cancer more extended D3 dissection has also been attempted to improve oncologic outcomes. Existing literature pertinent with the provisional therapeutic impact of D3 lymphadenectomy in advanced gastric cancer is studied in this mini review. Seven non-randomized comparisons, three randomized trials and five meta-analyses, almost exclusively of Asian origin, were identified and examined. D3 compared to D2 lymphadenectomy consistently and significantly proved to be associated with a “heavier” iatrogenic surgical trauma translated to more blood loss, prolonged operative time, higher relaparotomy rates and post-procedural surgical and non-surgical morbidity. Oddly mortality in most of these series did not reach statistical significance a fact probably attributed to Asian surgical expertise and/or methodologic drawbacks. All existing evidence and their meta-analyses, including a well-designed RCT from Japan (JCOG), failed to support a clear overall survival benefit linked to D3 dissection thus excluding the procedure from current treatment algorithms. The Italian GC research group, analyzing their database, proposed tumor histology, macroscopic type, size and location as selection criteria for D3 dissection provided surgical expertise is available. Recently, a phase II clinical trial from Japan reported a 3 -year survival rate of 59% in patients with clinically involved para-aortic nodes treated with neoadjuvant chemotherapy followed by D3 lymphadenectomy, rekindled the issue. Future multicenter randomized trials should test the extend and after effect of lymphadenectomy in gastric cancer combined with modern chemotherapeutic agents in multimodal treatments.

## Introduction

Lymphadenectomy constitutes an inseparable component of gastric cancer surgery. Lymph node excision contributes to cancer burden reduction, thus inhibiting local-regional progression and lowering recurrence probability, but also drives the staging procedure by identifying patient’s N status (1). If lymphadenectomy has an inherent therapeutic after effect remains a controversial issue. Updated version of gastric cancer staging system premises a min of 16 nodes to determine N parameter. The level of dissection which reproducibly ensures this numeric requirement has been described and nowadays consented as D2. D2 is also the globally agreed and recommended level of lymphadenectomy which promises optimum locoregional control and/or a provisional survival benefit (2). Confining or extending the level of lymphadenectomy predictably reduces or enhances morbidity and mortality respectively but does not modify disease progression or survival rates accordingly. Patients with early stages seem to benefit less from more extended lymphadenectomy, whereas patients with advanced stages also seem not to redeem any advantage given the high probability of disease’s systemic spread. Maximum benefit, by means of locoregional control and/or survival, is attributed to “middle staggers” (3). Attempts to dissect beyond the D2 standard level, aiming at additional disease control and survivorship have not been supported by hard evidence and were linked with higher morbidity. However, pertinent data from centers of expertise in gastric cancer surgery with D3 experience, report comparable to D2 morbidity and mortality rates. Some authors also attempted to specify a subgroup of patients who might benefit from D3 lymphadenectomy. Existing evidence supporting this perspective will be critically analyzed in this mini review.

## Background - Nomenclature

Letter D has been utilized to describe lymphadenectomy and Arabic numbers 1,2,3 three “dissection levels” in a scalable conception. Regional nodes draining stomach are grouped in “stations” numbered from 1 to 16. Furthermore, groups are categorized in three wider compartments described with capital letter N (N1, N2, N3). Corresponding levels of lymphadenectomy delimitated by these compartments are defined as D1, D2 and D3 ectomies respectively. It has been argued that when D > N, recurrence can be decreased. A gastrectomy which is bounded by compartment N2, is defined as a D2 gastrectomy and is widely accepted as the standard procedure of therapeutic intend in gastric cancer. All other than D2 ectomies are considered as non-standard procedures. D3 is defined as D2 +stations 13–16 (peripancreatic, superior mesenteric, meso-colic and para-aortic) (4). According to the latest TNM described in AJCC manual (8th edition), involvement of these stations are categorized as metastasis (M1) (5).

## Reasoning Supporting D3/extended Lymphadenectomy

Based on a simplistic model of centrifugal, stepwise, primarily lymphogenic cancer cell spread process, surgeons claimed that including in surgical specimen the outermost technically possible remote involved nodes might intercept cancer process. In this scenario, if the outermost nodes were by coincidence the terminal involved nodes at time of surgery, their excision would imply absolute radicality and highest probability for cure. If not, extended resection would lessen cancer burden, remove microscopic deposits and hopefully delay disease progression, lower recurrence rates and prolong disease free survival. In advanced stages microscopic metastatic involvement in para-aortic nodes was reported to be 6–33% hence identifying them as a “surgical target” (6). Additionally, the higher the number of nodes removed the more accurate the staging would be, eliminating thus “stage migration phenomenon” which hindered clear prognostication (7, 8). Single center cohorts of D3/extended lymphadenectomies reported 5 year survival rates of 12–23% supporting further feasibility and effectiveness investigation in the context of randomized controlled trials (9, 10).

## Comparative Evidence for Lymphadenectomy Beyond D2 Level

### Cohorts and Non-Randomized Trials

Tokunaga et al. (11), studied retrospectively the role of D3 lymphadenectomy in a series of 173 curatively resected patients with involved para-aortic lymph nodes (PALN) and reported a remarkable 5 year survival rate of 28,6% in subjects with less than 15 +ve nodes and any macroscopic type except Bormann 4. Thus, they suggested that D3 resection might be beneficial in selected PALN positive patients with no other non-curative factors, operated by adequately trained surgeons.

Maeta et. al (12) published the results of a single center, non RCT, comparing 35 “D4” patients (D3 +para aortic nodes) with 35 “D3” patients (D2 +peripancreatic, mesocolic, hepatoduodenal nodes), reporting higher morbidity, mortality, operation time and blood loss in “D4” group whereas mortality coincided. Authors speculated that a group of patients may benefit from D4 and survive longer and on this basis proposed a nationwide survey.

 Another non RCT from Ankara, Turkey compared 34/134 patients who underwent D3 gastric resection with 100/134 who underwent D2 resection. The overall operative mortality rate of D2 was 1% compared with 8,8% of D3 dissections (*p* < 0,05). Although the re-operation percentage was twice in D3 dissected patients, (11.8 vs. 6%), this difference did not reach statistical significance. D3 gastric resection was also linked with significantly increased morbidity (35.3 vs. 10%, *p* < 0,05). The authors postulated that D3 lymphadenectomy might have a role in advanced stages for which addable surgical morbidity and mortality are judged to be outmatched by contingent oncologic gain for this subgroup of patients (13).

A multicenter non RCT from Japan compared surgical results of 430 D2 gastrectomies with 150 D3 (14). Operation times (*p* < 0.0001), need for blood transfusion (15.1 vs. 53.3%; *p* < 0.001) and pulmonary complications (*p* < 0.001) were significantly lower in D2 group of patients. There was no significant difference in mortality rates and in overall and disease specific survival. Authors defined a subgroup of patients with tumor diameter between 50 and 100 mm that experienced statistically significant longer survival and lower locoregional recurrence rates. Their final conclusion was that D3 lymphadenectomy could be beneficial exclusively in tumors sized 50 to 100 mm or with pN1 stage, redeeming survival advantages for pN2 subjects exclusively among patients of the former group. Nevertheless, D3 lymphadenectomy in pN0 subjects or tumors <50 mm did not improve survival. Furthermore, in patients with multiple lymph node metastasis or tumors sized >100 mm, D3 dissection proved vain regarding survival.

A single center non RCT from China compared 66 patients who underwent D3 dissection with 55 who underwent D2 (15). By means of statistics no difference was detected among D3 and D2 groups regarding morbidity, blood loss, length of hospital stay, and mortality. Furthermore, the 3 year and 5 year overall survival rates reported in this series were not significantly different between D3 and D2 groups, being 77.5 vs 73.2% (*p* = 0.618), and 65.8 vs 66.1%, (*p* = 0.946) respectively. Thus, there was not overall survival benefit of D3 over D2 lymphadenectomy in this study. Logistic regression analysis linked PALN metastasis to metastasis of No. 8a and No. 9 lymphatic stations (*p* = 0.021 and *p* = 0.030, respectively) pointing out these nodal stations as provisional indicators for D3 dissection. Authors concluded that although D3 can be performed safely, it is not superior compared to D2 dissection regarding survival and thus should not be recommended routinely.

The Italian Research Group for Gastric Cancer (GIRCG) retrospectively reviewed its database to evaluate the impact of D3 lymphadenectomy on patterns of recurrence. Histology was identified as a significant determinant in the correlation between recurrence and extend of lymphadenectomy (*p* < 0.007). Higher recurrence rates were observed after D3 than after D2 dissection (45.1 vs 35.3%) in patients with intestinal type adenocarcinoma while the opposite was recorded in cases of mixed/diffuse histology (48.3 vs 61.5%). They suggested that this was due to the lymph-tropism characterizing diffuse histotype. Based on the above, this Italian group suggested D3 as a useful modification of standard D2 dissection addressed to advanced tumors of diffuse histology (16). Gastric upper third tumor location and T3-T4 depth of invasion were recognized as separate risk factors for PALN involvement and defined as selection criteria for D3 resection (17).

Recently, in a phase II trial launched by JCOG (Japan Clinical Oncology Group), gastric cancer patients with extensive involvement of regional (N2) nodes and/or para-aortic lymph node (PALN) metastases were treated with S-1 plus cisplatin in neoadjuvant setting followed by extended D3 surgery focusing in PALN dissection. Overall survival rates completing 3 and 5 years of follow up were 59 and 53%, respectively (18). These impressive results, support extended D3 lymphadenectomy after neoadjuvant chemotherapy as an auspicious treatment plan for patients with extensive nodal involvement in N2 tier-compartment and/or radiographically depicted PALN metastases (19).

### Randomized Controlled Trials (RCTs)

The Polish Gastric Cancer Study Group (PGCSG) launched a multicenter RCT initiated to evaluate the effects of D3 vs D2 lymph node dissection (20). Overall survival was defined as primary end point whereas morbidity, mortality, disease free survival and quality of life as secondary ones. Morbidity was insignificant between D2 (27.7%) and D3 (21.6%) groups (**p* = 0*.248). The same was true for postoperative mortality (4.9% for D3 vs 2.2% for D2; **p* = 0*.376). Authors specified splenectomy, pancreatic resection, blood loss >800 ml and cardiac disease as independent risk factors augmenting morbidity. The interim safety analysis revealed an insignificant difference regarding extent of lymph node dissection. There was not a different surgical outcome between extended and standard lymphadenectomy. Unfortunately, no survival data were reported.

The Japanese Clinical Oncology Group (JCOG), randomized 523 patients with resectable gastric cancer either to D2 dissection (263 patients) or to D3 dissection (261 patients). Overall survival was defined as the primary end-point. Incidence of operation-related complications were similar and insignificant between the two groups (20.9% for D2 and 28.1% for D3; *p* = 0.07). Also, thirty-day postoperative mortality of any cause did not differ significantly among the two groups (0.8% in both D3 and D2 group). The 5 year overall survival rate was similar for D2 and for D3 dissections (69.2 vs 70.3%); the hazard ratio for death was 1.03 (95% CI, 0.77 to 1.37; *p* = 0.85). Identically, recurrence-free survival was insignificant between the two groups; the hazard ratio for recurrence being 1.08 (95% CI, 0.83 to 1.42; *p* = 0.56). Based on these results authors concluded that D3 lymphadenectomy offers no additional survival advantage compared to that of standard D2 dissection in potentially curable gastric adenocarcinoma (21).

At the same period, the East Asia Oncology Group (EAOG) conducted a multicenter RCT of D3 vs D2 gastrectomy allocating 134 patients in each group (22). Overall survival was not significantly different between the D2 and D3 groups (*p* = 0.801). Blood loss and need for transfusion, morbidity and operation time were significantly different and higher in the D3 lymphadenectomy group compared to D2 group. Although statistically insignificant, postoperative mortality was higher in the D3 group than in the D2 group. Authors warned that D3 lymphadenectomy is a perilous operation to be undertaken only by trained and experienced surgeons. They also suggested D2 lymphadenectomy as the standard of care for potentially curable gastric cancer and rejected D3 dissection as risky and oncologically inefficient.

### Meta-Analyses

Wang Z et al. published a systematic review of the literature until 2009, including 2021 patients (4 RCT and 4 non-RCT) and concluded that extended lymphadenectomy (D2 +PAND): (a) when performed by experienced surgeons in high volume hospitals is equally safe to standard D2 dissection with low mortality, (b) by definition results in a higher “wound degree of surgery” translated to longer duration of operation and greater blood loss, (b) it does not improve overall survival of patients with advanced GC (23).

Lustosa SA et al. included in their meta-analysis two RCT (24, 25) concluding that D3 compared to D2/D1 added no survival benefit, and was linked to higher morbidity and prolonged operative times. All comparisons did not reach statistical significance (26).

Yang et al. meta-analyzed five RCT and three non-RCT, seven from Asia and one from Poland, including 1,452 patients of advanced stages. Operative mortality was 2,3% compared to 2,2% for D3 and D2 respectively (OR 1.05, 95% CI 0.49–2.27, *p* = 0.90). Postoperative morbitity was 24.7% compared to 29,6% (OR 0.78 CI 0.61–1.01, *p* = 0.06). Operative time and length of hospital stay were insignificant between D3 and D2 groups (*p* = 0.02 and *p* = 0.27 respectively). No survival benefit in favor of D3 dissection was reported (27).

A meta-analysis contacted by Chen XZ et al. studied three RCTs conducted by prestigious scientific groups: EASOG, JCOG, and PGCSG. Analysis failed to attribute a survival effect to D3 lymphadenectomy (RR 1.03; 95% CI, 0.93–1.14, *p* = 0,62). It was also indicated that D3 might not increase in-hospital or 30 day postoperative mortality rate (RR 1.03; 95% CI, 0.43–2.46; *p* = 0.95), but it tended toward increasing morbidity rate (RR 1.19; 95%; CI, 0.83–1.71; *p* = 0.35). Relaparotomy rate and need for blood transfusion were also higher in D3 groups (28).

The most up-to-date meta-analysis of D3 versus D2 lymphadenectomy included three RCTs from Japan (12, 21, 22). None of these three RCTs nor their meta-analysis could demonstrate a significant and robust association between overall survival and extend of lymph node dissection (HR 0.99, 95% CI 0.81–1.21). The one and only RCT which reported disease free survival (DFS) (21) failed to show any significant interaction between extend of lymphadenectomy and DFS (HR 1.08, 95% CI 0.83–1.42). Meta-analysis of these three trials also reported an insignificant difference regarding post-operative mortality between D3 and D2 groups (RR 1.67, 95% CI 0.41–6.73) (29).

## Discussion

The concept of extended lymphadenectomy in oncologic surgery developed during a period where other treatment modalities, - such as chemotherapy and radiotherapy-, were undeveloped or associated with excessive toxicity. Surgery carried the one and only chance for cure and palliation.

**Figure 1:**
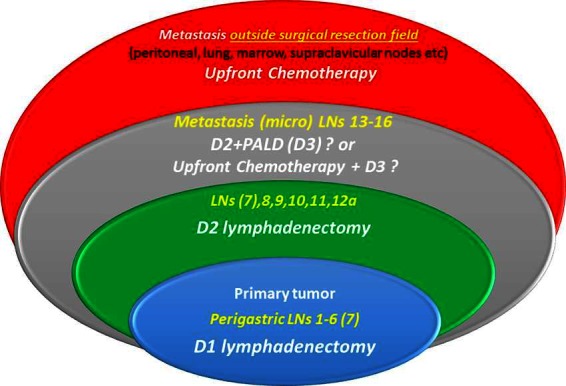
Micro-metastatic involvement of para-aortic nodes is considered borderline resectable disease, (gray ellipse), and could either be eliminated by surgery (D3 dissection) or sterilized with neoadjuvant chemotherapy and then resected by surgery. Macrometastatic involvement of distant nodes (stations 13–16) is classified as M overriding surgery’s endogenous therapeutic potential.

D3 lymphadenectomy consists a more radical operation expanding three-dimensional surgical resection margins, to augment the intrinsic therapeutic potential of surgical treatment. D3, by definition, aims to resect apart from tier 2 nodes those also contained in tier 3 (distant nodes = M1). Paraaortic lymph nodes are sites of metastatic spread in up to 20% of subjects with advanced resectable cancer (30) either as micro or as macro metastasis. Micrometastatic form might be considered as borderline resectable disease and a rather realistic surgical challenge whereas macro-metastasis a chemotherapy target with the hope of response and conversion to resectability state (Figure 1).

D3 dissection is indisputably a more technically demanding and complicated procedure compared to D1 or D2 as it requires dissection around large vessels located in deep retroperitoneal space. The more extended the dissection the more severe surgical injury and stress. Widespread use of modern hemostatic devices decreased operating time, surgical mortality, and procedural related morbidity. Available data suggest that D3 can be performed as safe as D2 procedures in the environment of high volume specialized centers by adequately trained surgeons even in western hemisphere (31).

The thorough literature search (Table 1), could not support superiority of D3 versus D2 lymphadenectomy concerning overall survival. However, the following limitations should be considered. No data on other survival endpoints (i.e., DSS or DFS) were available except in three papers (14, 21, 29). Moderate quality of evidence probably hindered a possible difference in postoperative mortality between D3 and D2. The methodological quality of pertinent studies was only moderate to poor (27). Other additional to surgery treatment modalities, such as chemo or radiotherapy, were scarcely reported in most of the studies (18, 21). Matching of groups with respect to clinical features, medical risk factors and tumor stage often was not well balanced (11, 13, 20, 22). In some RCTs reliable conclusions could not be reached due to small sample sizes(12, 13, 15). There was a heterogeneity regarding definitions of D2, D3 while other authors used terms such as D4 or D2 +PALN, thus producing a turmoil in comparisons. This may be attributed to term modifications through time while Japanese guidelines developed. Patient fitness for surgery and obesity two parameters which differentiate western from eastern patients may prevent even the most experienced surgeon to perform optimal by the book lymphadenectomy. Until today studies comparing D3 and D2 lymphadenectomy have been launched mainly in Asian subjects thus extrapolation of the results in Caucasians could be misleading.

**Table 1:** Details and outcomes of studies included in the mini review.

**Study reference/origin**	**Type-methodology**	**Patient selection and Interventions**	**Results**	**Comments**
Tokunaga M, 2010, Japan (11)	Retrospective cohort, Single center (*n* = 178)	“curative resection” PALN dissection	Morb:30%, Mort: 2%, 5yOS:13%Macroscopic type and number of positive nodes independent risk factors	**PALN might be beneficial in patients with <15 + ve nodes or macro type other than Bormann 4**
Maeta M, 1999, Japan (12)	Prospective, pilot not randomized, single center (*n* = 70)	T3–T4 tumors, normal nodes in CTD3 vs D4	D3 morb:26% D4 morb:40% SSFU ≤ 30 monthsOS NS	**D4 (no 16) dissection in pts with T3–T4 tumors and normal nodes in imaging did not improve survival and was linked to increased morbidity**
Bostanci E, 2004, Turkey (13)	Retrospective cohort, not randomized, single center (*n* = 134)	D2 vs D3	D2 Morb: 10% D3 Morb: 35% Morbidity SSD2 Mort: 1% D3 Mort :8% Mortality SS	**D3 can be performed with acceptable safety and might be an option for fit patients with potentially curable advanced disease**
Kunisaki C, 2006, Japan (14)	Retrospective comparison, not randomized, multicenter (*n* = 580)	T4 tumors (beyond sub-serosa)D2 vs D3	Morbidity NS (except bleeding, pulmonary and renal complications)Mortality NS	**No difference in OS and DSS. D3 might be advantageous in pts with pN2 and tumors sized 50–100 mm, in terms of DSS and recurrence**
Hu J, 2009, China (15)	Retrospective comparison, not randomized, single center (*n* = 117)	D2 vs D2 + PALN	D2 Morb:24,2% D2 + Morb:27,3% Morbidity NS D2 mort: 0% D2 + mort 1,8% Mortality NS Survival (5y): 65,8% vs 66,1% NS	**D2 + PALN is a safe procedure in experienced hands but offers no survival advantage and cannot be implemented in current recommendations**
De Manjoni G, 2011 and 2015, Italy (16, 17)	GIRGC retrospective database analysis, observational, multicenter (*n* = 568)	D2 vs D3	–	**Extend of lymphadenectomy had no impact in relapse. Pts with T3-4, with mixed/diffuse histology and upper third location might benefit from D3 dissection**
Tsuburaya A, 2014, Japan (18)	JCOG observational, multicenter (*n* = 53)	“bulky” pN2 and or PAN + in imagingS1 + cisplatin (4 weeks) followed by D2 + PALN	OS (3y): 59% OS (5y): 53%Grade3/4 toxicity: 34.4%	**For pts with “obvious” nodal involvement, neoadjuvant chemotherapy with S1/cisplatin followed by D2 + PALN is safe and occasionally effective**
Kulig J, 2007, Poland (20)	PGCSG, pilot, RCT, multicenter, (*n* = 275)	D2 vs D2 + PALN	Interim safety analysisMorbidity: 27,7 vs 21,6% NSMortality: 4,9 vs 2,2% NS	**Risk factors “fueling” complications were excessive blood loss, cardiac disease and splenectomy. No survival data reported**
Sasako M, 2008, Japan (21)	JCOG, multicenter, RCT (*n* = 523)	T2b, T3, T4 and “not obvious + PALN nodes”D2 vs D2 + PALN	OS (5y): 69,2 vs 70,3% NSDFS (5y): 62,6 vs 61,7% NS	**D2 + PALD compared to D2, offers no overall or recurrence free survival advantage in cT2b-T4, cPALN(−) pts**
Yonemura Y, 2008, Japan, Taiwan, Korea (22)	EASOG, multicenter, multinational RCT (*n* = 269)	Pts with enlarged PALN at CT excludedD2 vs D3	Mortality: 0,74 vs 3,73% NSOS (5y): 52,6% vs 55% NS	**D3 compared to D2 lymphadenectomy offers no significant survival advantage**
Wang Z, 2010, China (23)	Meta-analysis 4RCT, 4nonRCT trials (*n* = 2021)	D2 vs D2 + PALN	OS (5y): RR 0.96 vs 1.04 NS Mortality: RR 0.99 vs 2.06 NS	**D2 + PALD is a safe operation but without any survival benefit compared to D2 dissection**
Lustosa S, 2008, Brazil (26)(Asian patients)	Meta-analysis 5RCT D1vsD2vsD3 of which 2RCT D1vsD3 (*n* = 276)	D1 vs D3	Morbidity RR 2.35 vs 4.07 SS OS 5(y): RR 0,83 vs 1,38 NS	**D3 compared to D1 resulted to prolonged hospital stay, significant morbidity and mortality, no impact in 5 year survival**
Yang S, 2009, China (27)	Meta-analysis 5RCT (*n* = 1187)	D2 vs D3	Morbidity: 24,7 vs 29,6% NS Mortality: 2,3 vs 2,2% NS	**D3 does not offer any survival benefit and could increase the risk of surgical and non-surgical complications**
Chen X, 2010, China (28)	Meta-analysis 3RCT (*n* = 1067)	D2 vs D2 + PALD	Morbidity *p* = 0.05 SS Mortality *p* = 0.95 NS OS 5(y) *p* = 0.62 NS	**D2 + PALD is linked with increased morbidity and insignificant survival gain**
Mocellin S, 2015, Italy (29)(Asian patients)	Meta-analysis 3RCT (*n* = 862)	D2 vs D3	OS 5(y) *p* = 0.92 NS Mortality *p* = 0.57 NS	**No impact of D3 on overall or DFS survival, equally safe with D2 in expert’s hands**

Concluding remarks of each study are typed in bold text and listed in the outer-right column.

pts, patients; morb, morbidity; mort, mortality; DFS, disease free survival; DSS, disease specific survival; GIRGC, Group of Italian Research in Gastric Cancer; JCOG, Japanese Clinical Oncology Group; PGCSG, Polish Gastric Cancer Study Group; PALN/D, para-aortic lymph nodes/dissection; OS, overall survival; NS, non-significant; SS, statistically significant; RR, risk ratio.

Attempts to define separate prognostic factors to select patients who might benefit from D3 lymphadenectomy failed to establish a solid indication and modify treatment algorithm. Proximal tumor location, tumor diameter 50–100 mm, macroscopic type Bormann 4, T3–4 depth of invasion, pN1 disease, diffuse histology and involvement of nodes 8^α^ and 9 were all suggested as selective indicators for D3 lymphadenectomy (17). The most well conducted high quality RCT which addressed the question of provisional survival benefit of D3 vs D2 lymphadenectomy (JCOG-9501) failed to establish robust selecting criteria to define a subgroup for D3 (21). Following this, D3 is no longer defined in the latest Japanese-guidelines. Western surgical communities were eager to follow this recommendation and were rather relieved from the idea of a complex operative technique which had never implemented in their practice. The Italian Gastric Cancer Oncology Group published their D3 experience which coincided with this from Japan (32, 33).

 Notwithstanding D3 was due to fail in obscurity, it was recently brought out to spotlight again after encouraging survival rates observed in patients with clinically involved para-aortic nodes treated with neoadjuvant chemotherapy prior to resection. The JCOG conducted a phase II trial utilizing neoadjuvant chemotherapy followed by D3 dissection selecting patients with radiologically positive para-aortic nodes. The concept was successful: two cycles of S-1/cisplatin before surgery translated to a 5 year survival rate of 57% (19). This algorithm consists the tentative approach, included in the 4th version of the Japanese Gastric Cancer Treatment Guidelines in the section of clinical questions, and its perspective is to intensify neoadjuvant arm either by newer agents or by prolonging chemotherapy plan (34).

Further trials should be launched to test whether a more extended lymphadenectomy can be synergistically combined with adjuvant/neoadjuvant treatments to mutually enhance their therapeutic potential and thus extend survival. Modern chemotherapeutic agents might make extensive lymphadenectomy futile or vice versa (35).

Available evidence cannot support D3 lymphadenectomy as advantageous practice for the surgical treatment of resectable advanced gastric cancer. D2 lymphadenectomy is for the time being the recommended extend of nodal excision in gastric cancer surgery. Non-anatomic lymphadenectomy yields an unpredictable and often imperfect number of nodes and is unacceptable in the context of oncologic surgery. Comprehension and consolidation of D2 concept lymphadenectomy in western surgical training consists a high priority. Future trials should test after effect of lymphadenectomy in conjunction with novel chemotherapies.

## Author Contributions

GD project development, data collection, manuscript writing, and review manuscript. SP review the manuscript

## Conflict of Interest Statement

The authors declare that the research was conducted in the absence of any commercial or financial relationships that could be construed as a potential conflict of interest.

## References

[1] Dudeja V Habermann EB Abraham A Zhong W Parsons HM Tseng JF Is there a role for surgery with adequate nodal evaluation alone in gastric adenocarcinoma? J Gastrointest Surg (2012) 16(2):238–47. 10.1007/s11605-011-1756-7 22089951

[2] Degiuli M de Manzoni G di Leo A D'Ugo D Galasso E Marrelli D Gastric cancer: Current status of lymph node dissection. World J Gastroenterol (2016) 22(10):2875–93. 10.3748/wjg.v22.i10.2875 26973384 PMC4779911

[3] Hartgrink HH van de Velde CJ Putter H Bonenkamp JJ Klein Kranenbarg E Songun I Extended lymph node dissection for gastric cancer: who may benefit? Final results of the randomized dutch gastric cancer group trial. J Clin Oncol (2004) 22(11):2069–77. 10.1200/JCO.2004.08.026 15082726

[4] Japanese Gastric Cancer Association. Japanese classification of gastric carcinoma: 3rd English edition. Gastric Cancer (2011) 14(2):101–12. 10.1007/s10120-011-0041-5 21573743

[5] Amin MB AJCC Cancer Staging Manual. Springer International Publishing (2017). ISBN 978-319-40617-6

[6] Jiang BJ Gao YF Sun RX Shen H Lu M Cl T Clinical study on the dissection of lymph nodes around abdominal aortic artery in advanced gastric cancer. Zhongguo Putong Waike Zazhi (2000) 9:292–5.

[7] Smith DD Schwarz RR Schwarz RE Impact of total lymph node count on staging and survival after gastrectomy for gastric cancer: data from a large US-population database. J Clin Oncol (2005) 23(28):7114–24. 10.1200/JCO.2005.14.621 16192595

[8] Gholami S Janson L Worhunsky DJ Tran TB Squires MH Jin LX Number of lymph nodes removed and survival after gastric cancer resection: an analysis from the US gastric cancer collaborative. J Am Coll Surg (2015) 221(2):291–9. 10.1016/j.jamcollsurg.2015.04.024 26206635 PMC4654942

[9] Isozaki H Okajima K Fujii K Nomura E Izumi N Mabuchi H Effectiveness of paraaortic lymph node dissection for advanced gastric cancer. Hepatogastroenterology (1999) 46(25):549–54. 10228860

[10] Baba M Hokita S Natsugoe S Miyazono T Shimada M Nakano S Paraaortic lymphadenectomy in patients with advanced carcinoma of the upper-third of the stomach. Hepatogastroenterology (2000) 47(33):893–6. 10919056

[11] Tokunaga M Ohyama S Hiki N Fukunaga T Aikou S Yamaguchi T Can superextended lymph node dissection be justified for gastric cancer with pathologically positive para-aortic lymph nodes? Ann Surg Oncol (2010) 17(8):2031–6. 10.1245/s10434-010-0969-4 20182811

[12] Maeta M Yamashiro H Saito H Katano K Kondo A Tsujitani S A prospective pilot study of extended (D3) and superextended para-aortic lymphadenectomy (D4) in patients with T3 or T4 gastric cancer managed by total gastrectomy. Surgery (1999) 125(3):325–31. 10.1016/S0039-6060(99)70244-8 10076618

[13] Bostanci EB Kayaalp C Ozogul Y Aydin C Atalay F Akoglu M Comparison of complications after D2 and D3 dissection for gastric cancer. Eur J Surg Oncol (2004) 30(1):20–5. 10.1016/j.ejso.2003.10.008 14736518

[14] Kunisaki C Akiyama H Nomura M Matsuda G Otsuka Y Ono H Comparison of surgical results of D2 versus D3 gastrectomy (para-aortic lymph node dissection) for advanced gastric carcinoma: a multi-institutional study. Ann Surg Oncol (2006) 13(5):659–67. 10.1245/ASO.2006.07.015 16538414

[15] Hu JK Yang K Zhang B Chen XZ Chen ZX Chen JP D2 plus para-aortic lymphadenectomy versus standardized D2 lymphadenectomy in gastric cancer surgery. Surg Today (2009) 39(3):207–13. 10.1007/s00595-008-3856-x 19280279

[16] de Manzoni G Verlato G Bencivenga M Marrelli D di Leo A Giacopuzzi S Impact of super-extended lymphadenectomy on relapse in advanced gastric cancer. Eur J Surg Oncol (2015) 41(4):534–40. 10.1016/j.ejso.2015.01.023 25707350

[17] de Manzoni G di Leo A Roviello F Marrelli D Giacopuzzi S Minicozzi AM Tumor site and perigastric nodal status are the most important predictors of para-aortic nodal involvement in advanced gastric cancer. Ann Surg Oncol (2011) 18(8):2273–80. 10.1245/s10434-010-1547-5 21286941

[18] Tsuburaya A Mizusawa J Tanaka Y Fukushima N Nashimoto A Sasako M Neoadjuvant chemotherapy with S-1 and cisplatin followed by D2 gastrectomy with para-aortic lymph node dissection for gastric cancer with extensive lymph node metastasis. Br J Surg (2014) 101(6):653–60. 10.1002/bjs.9484 24668391

[19] Kodera Y Kobayashi D Tanaka C Fujiwara M Gastric adenocarcinoma with para-aortic lymph node metastasis: a borderline resectable cancer? Surg Today (2015) 45(9):1082–90. 10.1007/s00595-014-1067-1 25366353

[20] Kulig J Popiela T Kolodziejczyk P Sierzega M Szczepanik A, Polish Gastric Cancer Study Group. Standard D2 versus extended D2 (D2+) lymphadenectomy for gastric cancer: an interim safety analysis of a multicenter, randomized, clinical trial. Am J Surg (2007) 193(1):10–15. 10.1016/j.amjsurg.2006.04.018 17188080

[21] Sasako M Sano T Yamamoto S Kurokawa Y Nashimoto A Kurita A, et al. D2 lymphadenectomy alone or with para-aortic nodal dissection for gastric cancer. N Engl J Med (2008) 359(5):453–62. DOI 10.1056/NEJMoa0707035 18669424

[22] Yonemura Y Wu CC Fukushima N Honda I Bandou E Kawamura T Randomized clinical trial of D2 and extended paraaortic lymphadenectomy in patients with gastric cancer. Int J Clin Oncol (2008) 13(2):132–7. 10.1007/s10147-007-0727-1 18463957

[23] Wang Z Chen JQ Cao YF Systematic review of D2 lymphadenectomy versus D2 with para-aortic nodal dissection for advanced gastric cancer. World J Gastroenterol (2010) 16(9):1138–49. 10.3748/wjg.v16.i9.1138 20205287 PMC2835793

[24] Robertson CS Chung SC Woods SD Griffin SM Raimes SA Lau JT A prospective randomized trial comparing R1 subtotal gastrectomy with R3 total gastrectomy for antral cancer. Ann Surg (1994) 220(2):176–82. 10.1097/00000658-199408000-00009 8053740 PMC1234357

[25] Wu CW Hsiung CA Lo SS Hsieh MC Chen JH Li AF Nodal dissection for patients with gastric cancer: a randomised controlled trial. Lancet Oncol (2006) 7(4):309–15. 10.1016/S1470-2045(06)70623-4 16574546

[26] Lustosa SA Saconato H Atallah AN Lopes Filho GJ Matos D Impact of extended lymphadenectomy on morbidity, mortality, recurrence and 5-year survival after gastrectomy for cancer. Meta-analysis of randomized clinical trials. Acta Cir Bras (2008) 23(6):520–30. 10.1590/S0102-86502008000600009 19030751

[27] Yang SH Zhang YC Yang KH Li YP He XD Tian JH An evidence-based medicine review of lymphadenectomy extent for gastric cancer. Am J Surg (2009) 197(2):246–51. 10.1016/j.amjsurg.2008.05.001 18722583

[28] Chen XZ Hu JK Zhou ZG Rui YY Yang K Wang L Meta-analysis of effectiveness and safety of D2 plus para-aortic lymphadenectomy for resectable gastric cancer. J Am Coll Surg (2010) 210(1):100–5. 10.1016/j.jamcollsurg.2009.09.033 20123339

[29] Mocellin S Mcculloch P Kazi H Gama-Rodrigues JJ Yuan Y Nitti D Extend of lymph node dissection gor adenocarcinoma of the stomach. Cohrane Database of Syst Rev (2015) 8:CD001964. 10.1002/14651858.CD001964.pub4 PMC7263417 26267122

[30] Takashima S Kosaka T Results and controversial issues regarding a para-aortic lymph node dissection for advanced gastric cancer. Surg Today (2005) 35(6):425–31. 10.1007/s00595-004-2976-1 15912287

[31] Roviello F Pedrazzani C Marrelli D di Leo A Caruso S Giacopuzzi S Super-extended (D3) lymphadenectomy in advanced gastric cancer. Eur J Surg Oncol (2010) 36(5):439–46. 10.1016/j.ejso.2010.03.008 20392590

[32] Verlato G Roviello F Marchet A Giacopuzzi S Marrelli D Nitti D Indexes of surgical quality in gastric cancer surgery: experience of an Italian network. Ann Surg Oncol (2009) 16(3):594–602. 10.1245/s10434-008-0271-x 19118437

[33] Marrelli D Pedrazzani C Neri A Corso G Destefano A Pinto E Complications after extended (D2) and superextended (D3) lymphadenectomy for gastric cancer: analysis of potential risk factors. Ann Surg Oncol (2007) 14(1):25–33. 10.1245/s10434-006-9063-3 17024558

[34] Kodera Y Sano T Japanese gastric cancer treatment guidelines 2014 (ver. 4). Gastric Cancer (2017) 20:1–19. 10.1007/s10120-016-0622-4 PMC5215069 27342689

[35] Jansen EP Boot H van de Velde CJ van Sandick J Cats A Verheij M Can adjuvant chemoradiotherapy replace extended lymph node dissection in gastric cancer? Recent Results Cancer Res (2012) 196:229–40. 10.1007/978-3-642-31629-6_16 23129378

